# A Machine Learning Approach to Minimize Nocturnal Hypoglycemic Events in Type 1 Diabetic Patients under Multiple Doses of Insulin

**DOI:** 10.3390/s22041665

**Published:** 2022-02-21

**Authors:** Adrià Parcerisas, Ivan Contreras, Alexia Delecourt, Arthur Bertachi, Aleix Beneyto, Ignacio Conget, Clara Viñals, Marga Giménez, Josep Vehi

**Affiliations:** 1 Institut d’Informàtica i Aplicacions, Universitat de Girona, 17003 Girona, Spain; adria.parcerisas.albes@gmail.com (A.P.); ivancontrerasfd@gmail.com (I.C.); alexia.delecourt@gmail.com (A.D.); aleix.beneyto@udg.edu (A.B.); 2 Campus Guarapuava, Federal University of Technology—Paraná (UTFPR), Guarapuava 85053-525, Brazil; abertachi@utfpr.edu.br; 3 Endocrinology and Diabetes Unit, Hospital Clínic, 08036 Barcelona, Spain; iconget@clinic.cat (I.C.); vinals@clinic.cat (C.V.); gimenez@clinic.cat (M.G.); 4 Centro de Investigación Biomédica en Red de Diabetes y Enfermedades Metabólicas Asociadas (CIBERDEM), 28029 Madrid, Spain; 5 Institut d’Investigacions Biomèdiques August Pi i Sunyer, 08036 Barcelona, Spain

**Keywords:** hypoglycemia, machine learning, multiple daily injections, prediction model, support vector machine, type 1 diabetes

## Abstract

Nocturnal hypoglycemia (NH) is one of the most challenging events for multiple dose insulin therapy (MDI) in people with type 1 diabetes (T1D). The goal of this study is to design a method to reduce the incidence of NH in people with T1D under MDI therapy, providing a decision-support system and improving confidence toward self-management of the disease considering the dataset used by Bertachi et al. Different machine learning (ML) algorithms, data sources, optimization metrics and mitigation measures to predict and avoid NH events have been studied. In addition, we have designed population and personalized models and studied the generalizability of the models and the influence of physical activity (PA) on them. Obtaining 30 g of rescue carbohydrates (CHO) is the optimal value for preventing NH, so it can be asserted that this is the value with which the time under 70 mg/dL decreases the most, with almost a 35% reduction, while increasing the time in the target range by 1.3%. This study supports the feasibility of using ML techniques to address the prediction of NH in patients with T1D under MDI therapy, using continuous glucose monitoring (CGM) and a PA tracker. The results obtained prove that BG predictions can not only be critical in achieving safer diabetes management, but also assist physicians and patients to make better and safer decisions regarding insulin therapy and their day-to-day lives.

## 1. Introduction

Type 1 diabetes (T1D) is a chronic condition resulting from the autoimmune destruction of insulin-producing β cells in the pancreas [1,2]. People suffering from this condition are treated with lifelong intensive insulin therapies. While these treatments allow patients to reduce the amount of blood glucose (BG), avoiding hyperglycemia [3,4,5] and reducing complications [1,5], they are burdened by the common side effect of over-lowering glucose which can drive the patient into hypoglycemia. Patients with T1D face the challenge of reducing hyperglycemia without causing hypoglycemic events, keeping blood glucose levels within a safe range [1,6,7,8]. Usually, the strongest variations in BG signals occur after meals and during the night [4,9,10], the latter variations being the main causes of postprandial hypoglycemia or nocturnal hypoglycemia (NH) [11,12].

The risk of NH is associated with several risk factors, such as previous episodes of hypoglycemia, low glycated hemoglobin (HbA1c), impaired hypoglycemia awareness, and increasing duration of T1D [1,13]. Additionally, several circumstances favor the development of NH such as excess/wrong dose of insulin, inadequate carbohydrate (CHO) intake, alcohol consumption, and previous physical activity (PA) [1]. Consequences of suffering an NH may include confusion, sweating, seizures, and even death [6,13]. These adverse events reduce health-related quality of life and work performance. In addition, the fear of suffering NHs is considered an important psychological factor which may undermine resting periods [14]. Thus, NHs may cause poor sleep and so reduce the quality of life of T1D patients [15].

In the last decade, efforts toward the development of an artificial pancreas, a closed-loop glucose monitoring system using subcutaneous glucose sensing, continuous subcutaneous insulin infusion from a pump, and a control algorithm have enabled advances in diabetes management, patient safety, and the prevention of glycemic events [6]. However, the fact that the implementation of these technologies is not adaptable to all patients with T1D, together with the high cost associated with this technology, prevents their generalization [16,17,18]. Therefore, therapies based on multiple daily injections (MDI), the combination of slow-acting insulin for basal coverage and rapid-acting insulin at mealtimes to control postprandial blood glucose levels, are still the most widespread treatments for people suffering from type 1 diabetes [16].

Technically, the incorporation of CGM into MDI generates a huge amount of useful data [16] that can be used to improve diabetes management. Based on the latter, and after the implementation of promising ML approaches to the continuous prediction of BG values and NH in sensor-augmented insulin pump (SAP) therapy [9,12], information from CGM and other sources have been used by ML algorithms to predict and prevent postprandial hypoglycemia [9,19] for patients using sensor-augmented pump and MDI therapies. Recently, ML algorithms have also been used to predict nocturnal hypoglycemia for insulin pump users [17,20,21]. More recently, the feasibility of personalized models to anticipate NH in 10 T1D patients was investigated. Patients underwent MDI therapy and, alongside data from CGM, insulin, and carbs, physical activity was also taken into account [1]. Results indicate that more than 70% of the NH events could be predicted and eventually avoided.

In this paper, a method to reduce the occurrence of NH is presented, providing a decision support system to people with T1D and improving self-confidence during the management of the disease. To this end, several improvements have been made to make the method useful in practice. First, the algorithms for predicting NH have been optimized for a reduced number of features. Second, population models have been developed and validated for a specific population. Third, the impact of PA functions on system performance has been investigated and lastly eliminated from the set of functions, so that prediction works using only information from CGM and MDI therapy, thus simplifying the overall system. Finally, a strategy to reduce NH is proposed and validated “in silico”.

## 2. Materials and Methods

### 2.1. Patients and Dataset

The study database includes 10 patients that were monitored for 12 weeks. The clinical trial was conducted at the Hospital Clinic de Barcelona and has been registered under the identifier NCT03711656 at ClinicalTrials.gov (accessed 2 February 2022). Inclusion criteria included being adult patients (over 18 years of age) with more than five years of diabetes and MDI treatment, HbA1c between 6.5% and 9.5%, and more than 4 hypoglycemia episodes per week. In addition, participation in the trial required basic knowledge to follow the study instructions, including the use of a CGM, the ability to estimate CHO, and to have a minimum of 4 blood glucose measurements per day. Exclusion criteria were pregnancy, a serious illness that could affect participation in the study, and any use of an experimental drug or device 30 days prior. Monitoring was performed under free-living conditions at home with a CGM and a physical activity monitor. Abbott FreeStyle Libre (Abbott Diabetes Care, Alameda, CA, USA) was used as the CGM system to monitor interstitial glucose concentration and a Fitbit Alta HR wristband (Fitbit, Inc., San Francisco, CA, USA) was used to obtain PA information and sleep periods.

### 2.2. Data Processing and Feature Engineering

**Figure 1:**
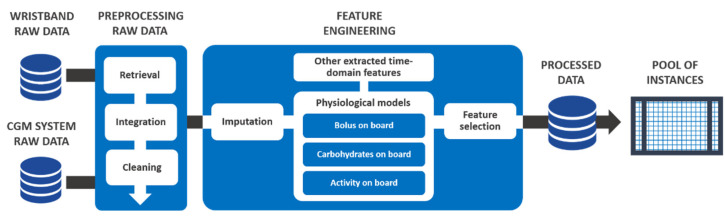
Methodology applied to prepare the raw data for machine learning.

The development of prediction models has followed an approach similar to that developed by Bertachi et al. [1,2]. ML techniques were applied to a pool of instances extracted from the collected data. Figure 1 illustrates the overall preparation process for obtaining these sets of instances. The variables collected from the CGM system were interstitial glucose concentrations, meal estimations, insulin bolus doses, and self-monitoring blood glucose measurements, while the variables collected by the wristband were heart rate signal, steps performed, estimation of calories burned, and sleeping period. All of these variables were collected with their respective timestamps. The data retrieved from the CGM and wristband database systems were integrated and cleaned.

Next, we applied a feature engineering phase, which encompasses tasks to provide additional value to the dataset: imputation of missing values, feature extraction, and feature selection. We performed a simple procedure of imputation of missing data, one in which linear interpolation was applied to those gaps in the CGM measurements that were equal to or shorter than 120 min. After that, we applied three physiological models to the data to obtain semi-continuous representation features of the effects of fast-acting insulin doses, estimated carbohydrates, and detected steps. First, we applied the bolus on board (BOB) model [11] that estimates the amount of insulin active in the body. Second, the CHO on board (COB) model [12] was applied in all the records of ingested meals. The COB, conceptually similar to BOB, represents the amount of CHO consumed that still has not appeared in plasma. Third, the activity onboard (AOB) model [19] represents the accumulated effects of PA in the body. Finally, a process to select the minimum number of features was undertaken in order to improve the model performance and its computational cost and execution time.

The 29 time-domain features extracted from the 6 h of data prior to the start of the patient’s sleep period and proposed by Bertachi et al. [1] were reduced to the 17 features without a significant loss of performance:

1. CGM [t]: BG value from CGM device at the time of prediction (t).
2. CGM mean [t, t − 60]: mean interstitial glucose measurements of the last hour.
3. CGM mean [t − 60, t − 120]: mean interstitial glucose measurements between one hour and two hours before sleeping period.
4. CGM mean [t − 120, t − 180]: mean interstitial glucose measurements between two hours and three hours before sleeping period.
5. CGM mean [t − 180, t − 240]: mean interstitial glucose measurements between three hours and four hours before sleeping period.
6. CGM mean [t − 240, t − 300]: mean interstitial glucose measurements between four hours and five hours before sleeping period.
7. CGM mean [t − 300, t − 360]: mean interstitial glucose measurements between five hours and six hours before sleeping period.
8. ∆BG [t, t − 30]: BG variability of the last half-hour.
9. ∆BG [t, t − 60]: BG variability of the last hour.
10. BOB: estimated active insulin in the body.
11. COB: estimated CHO in plasma.
12. AUC70: the total area of blood glucose levels below 70 mg/dL on the blood glucose curve of CGM during the last 6 h.
13. LBGI [t − 300, t − 360]: the low blood glucose index risk of BG variation during the last 6 h.
14. HBGI [t − 300, t − 360]: the high blood glucose index risk of BG variation during the last 6 h.
15. Rate of Change [t, t − 30]: the BG values variation during the last 30 min before sleeping period.
16. AOB: the accumulated effects of PA at bedtime.
17. Steps: daily steps number.

The labeling of the instances included the 6 h after the onset of the sleep period and considered three consecutive interstitial glucose values below 3.9 mmol/L (70 mg/dL) as an episode of hypoglycemia. Therefore, instances with these values were labeled as Class 1 (night with hypoglycemia) and in any other case were assigned Class 0 (night without hypoglycemia).

### 2.3. Performance Metrics

This study has used multiple metrics based on the confusion matrix to evaluate the performance of the methodology implemented. The positive (P) and negative (N) labels refer to the predicted outcome, while the true (T) and false (F) labels refer to the actual outcome. Table 1 presents the main metrics employed, defining sensitivity (SE), specificity (SP), Matthews correlation coefficient (MCC), F1 score, and Gmean. The MCC, F1score and Gmean were all calculated through the corresponding formulas expressed in Table 1. All of them were considered as each of them uses the results of the confusion matrix in a different way. While the F1 score ignores the count of true negatives, the MCC kindly extends its care to all four entries of the confusion matrix, while Gmean takes into account both previous metrics. In addition, the area under the curve (AUC) of the ROC curve is used throughout the study.

### 2.4. Algorithm Selection

The application of ML techniques for diabetes management has been largely explored [16]. Different initiatives have tried to establish a ranking between different approaches and there are noteworthy events like “BG Prediction Challenge” [22] that aims to compare the performance and appropriateness of the algorithms presented within an identical framework. However, there is not a conclusive answer on whether there is any algorithm, or set of them, that obtains a better overall performance in the task of generating models for the prediction of BG values and, in its extension, hyperglycemic and hypoglycemic events [12,13]. Hence, a preliminary study of a diverse set of ML techniques was conducted. This study aimed to determine the most suitable methodology for the generation of binary predictive models using a set of standard ML algorithms from the library scikit-learn of Python. The initial set includes the following methodologies: artificial neural network; multinomial naïve Bayes; adaptive boosting (AdaBoost); support vector machines (SVM); linear discriminant analysis (LDA) and long short-term memory (LSTM).

The preliminary study involved a series of tests of overall available instances to measure the performance of the proposed techniques. The pool of instances was divided into training data (80%) and test data (20%). The results of this preliminary study show the best results for the SVM algorithm. Therefore, this study has considered that SVMs are the most suitable option to implement more advanced features, which is in line with the algorithm selection of the previous articles [1,9].

### 2.5. Building Machine Learning Models

While the personalization of models has the obvious advantage of creating a custom model that is perfectly suited to the characteristics of a patient and recording device, it also has multiple disadvantages: (i) it limits the usability of the failure in that the system cannot be used on a patient until the data has been calibrated, (ii) it limits the generalization capabilities of the system and increases the risks of overfitting. Conversely, learning a model from a heterogeneous group of patients increases the robustness of the recording devices in principle [23]. Population models have the advantage of creating a common system for all users, and therefore reduce the burden of computing and give faster results. However, such patient variability severely limits the use of general models, which cannot capture the specific physiological behaviors of individuals [16].

This study aims to investigate the possibility of training a population model and the ability of ML techniques to cope with the lack of personalized data. Therefore, the next step was to generate population and individual models:

- Population models: The models of this batch of experiments use a leave-one-out scheme, thus involving all the patient data except data from one of them, which will be used later for testing. The general population model is useful to see what results would be obtained if the model were applied to a completely new patient. The process is similar to that used with the population models. In this case, the testing dataset for the validation of the model is from a specific patient to be evaluated. Then, a model is created for each patient.
- Personalized models: The customized model or personalized model is trained and validated with data from a specific patient, which is basically to create a model for that particular patient. The same steps are applied to design these models. The main and unique difference is that the data used for the implementation of each model is from a single patient. Thus, a model is generated for each patient as well.

**Figure 2:**
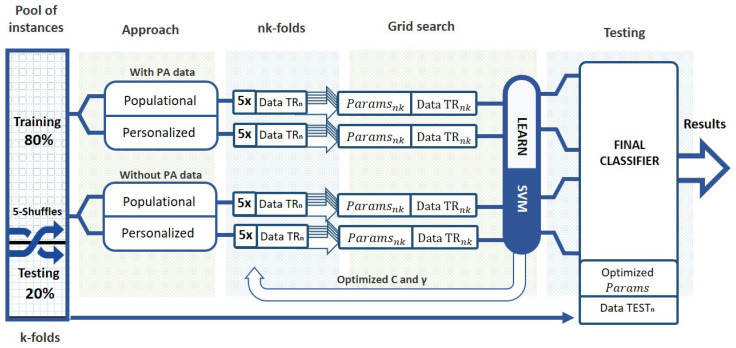
General diagram of the methodology employed to build the machine learning models.

For each of the cases, personalized and population, two models were trained, using the data of physical activity or its lack. The objective was to analyze if models that do not have information on PA and, therefore, require one less device, are precise enough to be used to avoid episodes of hypoglycemia. After selecting the data involved in each of the approaches, the procedure for building the models is the same for all four of them. As shown in Figure 2, the data was split into training (80%) and testing (20%) datasets using a nested fivefold cross-validation scheme to ensure the robustness of the model. The optimization of the hyperparameters (C and γ) of each prediction model was performed using a grid search with a stratified fivefold cross-validation. In this way, a range of C and γ values were tested and those who generated better results were selected. This process guarantees robustness and similar class distribution in each fold despite the great imbalance between classes. Finally, the model was validated with the previously divided test data set. From the five iterations, the median results were obtained for each of the metrics.

### 2.6. Mitigation Measures

**Figure 3:**
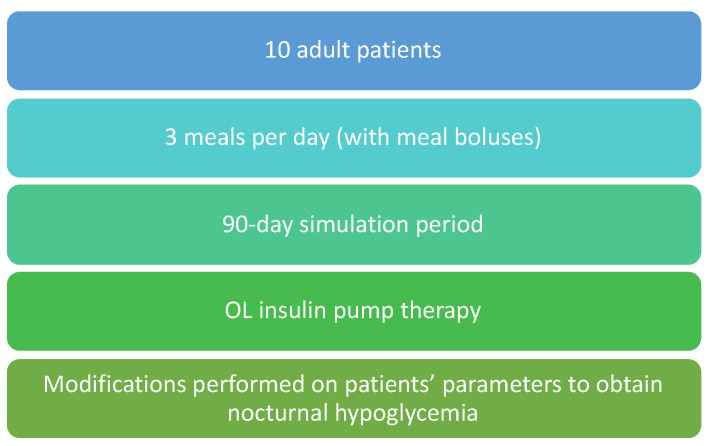
Summary of the parameters and implemented features in the UVA Padova simulator to validate the mitigation measures. Abbreviations: OL, open loop.

Once a population model has been developed, it is necessary to design mitigation measures to prevent and reduce the number of NH and evaluate in advance the impact they will have before conducting clinical trials. For this purpose, a modified version of the UVA Padova simulator [24] has been used. The modification undertaken was intended to generate a population mimicking our cohort of real patients (see Figure 3). The similarity considered the occurrence of nocturnal hypoglycemia between both cohorts. To resemble it, the parameters related to insulin sensitivity were modified (parameters VMX and kp3 from Dalla Man’s Model [24]). These parameters were modified manually only during an overnight period (between 00:00 and 06:00 in the simulations) to simulate nocturnal hypoglycemia.

In the sequence, the results of SE and SP obtained by the population model were considered in order to assess the mitigation actions. Mitigation actions consist in giving the patient a certain amount of rescue CHO to avoid the predicted glycemic drop during the night. Therefore, considering a random number (uniformly distributed) and the probability of a correct prediction (given by SE and SP), a certain amount of CHO is consumed by the patients. A bi-exponential absorption model such as the one from Hovorka et al. [25] has been applied to model the effects of such CHO. Related to this model, different time constants have been evaluated to determine the most suitable absorption rate constant (τ_max) for the type of snack the patient can consume when the mitigation action is required. A similar procedure has been carried out to determine the optimal quantity of carbohydrates. Finally, in order to determine if the results from both simulations are statistically significant from the baseline, a Wilcoxon signed-rank test has been conducted.

Apart from assessing the different reductions in the number of nights with hypoglycemia, it is important to evaluate the effects of rescue CHO on BG levels. To do so, the percentage of time in range in different glycemic intervals has been computed for each patient. The intervals selected for the evaluation are:

- Below 54: BG levels below 54 mg/dL, also known as level 2 hypoglycemia. This refers to clinically severe hypoglycemia.
- Below 70: BG levels below 70 mg/dL, also known as level 1 hypoglycemia. This refers to time below range (TBR) levels.
- 70–180: BG levels between 70 and 180 mg/dL, also known as time in range (TIR) levels.
- Above 180: BG levels above 180 mg/dL, also known as time above range (TAR).

For each of these intervals, the median and interquartile range have been computed for the baseline simulation, and for simulations with both 25 and 30 g of rescue CHO. Furthermore, the variation with respect to the baseline simulation, and the *p*-value from the corresponding Wilcoxon signed-rank test, have been calculated.

## 3. Results

A total of ten subjects completed the study. The average age was 31.8 (SD 16.8) years, the HbA1c 7.3 (SD 0.5) %, the body mass index 24.6 (3.6) kg/m^2^, and duration of diabetes 20.0 (SD 8.9) years. Among them, 8 (80%) were women. The median number of instances per patient was 67 (SD 28.2). NH occurred in approximately one third of the nights, 22 (SD 16.5).

### 3.1. Prediction Models Performance

Table 2 and Table 3 show the outcomes of the prediction models’ results including and excluding PA information respectively. Considering the median outcomes of SE and SP obtained for each model, it can be stated that there is not much difference between population and personalized models. In addition to this fact, the results for F1score and Gmean metrics in the case of the population model show superior values. Considering the median outcomes for all patients using this metric, almost 75% of NH would be predicted, achieving a median specificity of 77% and 68% in population and personalized models, respectively. For models excluding PA, better outcomes were obtained with models optimized with MCC.

**Figure 4:**
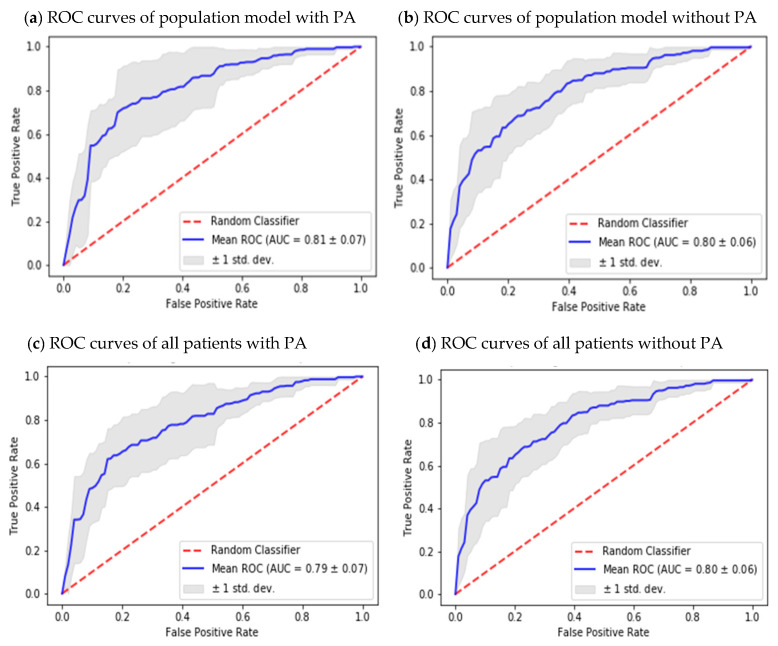
(**a**,**b**) represent the receiver operating characteristic (ROC) curves generated by population models, both including (left) and excluding (right) PA models. Figures (**c**,**d**) represent the ROC curves generated by population models per patient, both including (left) and excluding (right) PA models.

The best result was obtained for individual P56 achieving 95% of sensitivity and 75% of specificity. The worst outcomes were obtained for individual P12, showing a sensitivity of 39% and 80% of specificity. Population models without PA information were slightly inferior. In this case, the models obtained a median of almost 70% of sensitivity and a specificity of 73%. Best outcomes were achieved for individual P45 with 86% of sensitivity and 69% of specificity. The worst results were obtained for individual P12 achieving 42% of sensitivity and 75% of specificity. Considering population models, the median of ROC curves, including and excluding PA features, was calculated as well (see Figure 4). The results were 81 (SD 0.07) and 80 (SD 0.06), respectively. Regarding population models per patient using PA features, the median of ROC curves was 79 (SD 0.07). For the models excluding the PA variables, the median was 80 (SD 0.06). Results are also shown in Figure 4.

### 3.2. Reduction of Nocturnal Hypoglycemic Events

The solution to the minimization of the number of hypoglycemia events at night consists in recommending the patient consume a snack before going to bed in case the model predicts hypoglycemia. It is expected that this will lead to a reduction in the number of hypoglycemia events, at least early in the night, and also in the duration of the hypoglycemia.

Given that the patient is going to consume a specific type of snack, 20, 25, 30 and 35 g of rescue CHO were tested. It has been seen that with the introduction of rescue CHO there is actually a reduction in the number of NH events. However, the amount of CHO does not really seem to provide different results. This may be due to the variability of CHO absorption. For this reason, a consequent study was also conducted with different $\tau_{max}$. Results in Table 4 show that different time constants do not necessarily lead to better results. Given that a time constant of 20 min seemed too low to obtain a satisfactory absorption and a time constant of 60 min does not provide superior results, the first approach was to choose a $\tau_{max}$ of 40 min.

**Figure 5:**
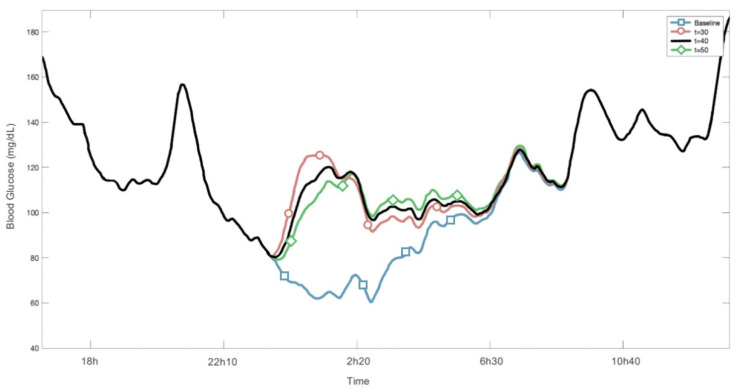
Example of action to prevent a hypoglycemic event with 30 g CHO. In blue: the baseline mg/dL of a given patient during the event. In red, black, and green the mg/dL when 30 g of rescue CHO were ingested with different time constants of absorption.

In order to test if it is the case here, the prevention of a specific hypoglycemic event with 30 g of CHO and a $\tau_{max}$ of 40 min along with a ±10 minutes’ variability has been plotted. As outlined by the plots in Figure 5, it can be seen that even with a variable $\tau_{max}$ the system is still capable of preventing the event from happening. The dotted line corresponds to the baseline simulation (i.e., without rescue CHO), which in this case delineates the hypoglycemic event (blood glucose below 70 mg/dL). On the other hand, the star, cross and square lines correspond to mitigation actions with 30 g of rescue CHO at t_0_=11:30 pm and $\tau_{max}$ = 30 min, $\tau_{max}$ = 40 min, and $\tau_{max}$ = 50 min, respectively.

Consequently, simulations were performed setting the simulator’s SE to 0.73 and SP to 0.75, employing a $\tau_{max}$ of 40 min and introducing 20 or 30 g of rescue CHO. Results are presented in Table 5 and demonstrate two things. First, since the *p*-values are both 0.002, we can assert that ingesting this amount of CHO will significantly reduce the occurrence of NH. Second, the dose of 30 g of rescue CHO provides slightly better outcomes than 20 g.

Regarding time in range (TIR), results are shown in Table 6. The value with which the time under 70 mg/dL decreases the most is 30 g, while increasing the time in the target range by 1.3%, confirming that 30 g is in fact the most appropriate value for preventing NH. What is more, and also with 30 g of CHO, level 2 NH is reduced by more than 40%. In addition, all the aforementioned facts show *p*-values lower than 0.05, which indicates a statistically significant difference with respect to the baseline simulation.

## 4. Discussion

In this article, different machine learning algorithms, data sources, optimization metrics and mitigation measures to predict and avoid nocturnal hypoglycaemic events have been studied. In addition, we have studied the generalizability of the models and the influence of physical exercise on them. As a main result, a population model capable of predicting more than 40% NH has been developed, converting the theoretical model in ref. [1] into a practical case because of the inclusion of mitigation measures.

As a preliminary study determined that the demographic data of the study population is not significant, these types of variables, such as sex and age, were not considered for the rest of the process. Once the ML methodology was selected, we studied the optimization metrics. The Gmean metric was adopted to select the best prediction model, not only for better results but also because it applies the same weight to the SE and SP metrics. Although the principal goal is NH avoidance, benefits should be balanced against potential side effects, such as false positives that may lead to unnecessary ingestion of CHO and high BG values.

Regarding the different models studied, the population model shows results sufficiently similar to the individual models. One of the main objectives was to implement a model that could be population-based rather than individual-based, not only to try to reduce the time burden on the algorithm, but also due to the scarcity of clinical data to perform sufficiently personalized individual models with optimal results. As the results of the population model study were significantly similar to those of the individual model study, the population model was applied to the simulator considering the limited database of the study and the previously mentioned benefits it brings. The simulation results have been encouraging as it has been observed that about 1/3 of NH could be avoided. With these results, we can corroborate that a more accurate prediction of hypoglycemia/hyperglycemic events can give rise to a better management of the disease in the short term, and make predictive models more reliable for both physicians and patients using MDI [9]. We have also carried out a study of the impact of PA information on the predictions, from which we have been able to extract that it is an important factor to consider for the development of hypoglycemia. The results have been better when PA features have been included, corroborating previous theories [1,7,13,18]. Thus, the monitoring devices and sensors for PA should be actively updated, as the inclusion of exercise-related signals in future modelling strategies constitutes a very important research opportunity [10].

Many other proposals [1,13,18,19] have been developed with no clinical evidence and only validated with in-silico data. Here, we have taken a further step in the validation procedure, implementing a simulator that uses a cohort of patients with similar conditions to the real group. The mitigation measures have been designed in such a way that if the model predicts an NH, the patient is advised to consume a certain amount of CHO. The statistical results from each simulation confirm that 30 g of CHO are in fact the most appropriate value for preventing NH. Given that 30 g of rescue CHO is the optimal value for preventing NH, we can assert that this is the value with which the time under 70 mg/dL decreases the most, with almost a 35% of reduction, while increasing the time in the target range by 1.3%.

It is also worth mentioning that a *τ_max_* of 40 min for the absorption of carbohydrates has been chosen because it seemed like the most appropriate absorption time for the type of snack that is advisable for the patient before going to sleep. Certainly, we need to be aware that before going to sleep, we cannot ask the patient to have another meal, but we still need a slow CHO absorption rate. Possible suggestions are a glass of milk with cookies or yogurt with sugar free biscuits.

In this study, we considered the limited number of instances as a study constraint. To the point that there is missing data or lack of a few instances. It is likely that, in a study with a longer follow-up period of the patients, improvements could be obtained in the models, particularly the personalized models. Our study included a focalized group of T1D patients particularly predisposed to NH. Therefore, we do not know if the results would apply to participants with a lower risk of nocturnal hypoglycemia. Finally, it could be considered that 30 g is a considerable, unpractical, and even unappetizing amount of CHO at night just to prevent a hypothetical hypoglycemia provoking an undesirable rise in BG values when a false positive prediction occurs.

## 5. Conclusions

In this paper, an algorithm to reduce the number of NH is presented, tested, and validated in-silico, providing a decision support system to people with T1D and improving self-confidence during the management of the disease. With this new tool, T1D patients with MDI therapy might be able to reduce more than a third of NH, improving the management of the disease and increasing their clinical safety. The results obtained in this study prove that BG predictions can not only be critical in achieving safer diabetes management, but also assist physicians and patients to make better and safer decisions regarding insulin therapy and their day-to-day lives.

In future works, a huge set of data is needed to fully validate the proposed approach. On the one hand, some techniques could improve their performance. On the other hand, more advanced classification techniques, such as deep learning, could also be evaluated. Also, a future improvement of the mitigation measures proposed may be undertaken in order to evaluate the absorption behavior of each of the snacks.

Future clinical trials are being prepared by our research group and should be conducted soon. In order to proceed with the clinical trials, we have already carried out a preclinical study and developed its respective protocol. The analytical models presented in this article will be implemented in a smartphone application that will support patients to avoid hypoglycemic episodes at night.

## Figures and Tables

**Table 1:** Equations of the performance metrics evaluated.

Performance Metrics	
$\mathrm{SE}=\frac{\mathrm{TP}}{\mathrm{TP}+\mathrm{FN}}$	$\mathrm{MCC}=2*\frac{\mathrm{TP}*\mathrm{TN}-\mathrm{FP}*\mathrm{FN}}{\sqrt{\left(\mathrm{TP}+\mathrm{FP}\right)\left(\mathrm{TP}+\mathrm{FN}\right)\left(\mathrm{TN}+\mathrm{FP}\right)\left(\mathrm{TN}+\mathrm{FN}\right)}}$
$\mathrm{SP}=\frac{\mathrm{TN}}{\mathrm{TN}+\mathrm{FP}}$	$F1\mathrm{score}=\frac{2*\mathrm{TP}}{2\mathrm{TP}+\mathrm{FP}+\mathrm{FN}}$
$\mathrm{Gmean}=\sqrt{\frac{1}{\mathrm{SE}*\mathrm{SP}}}$	

**Table 2:** Median sensitivity (SE) and specificity (SP) of the model using support vector machines (SVM), including physical activity measures. Three different performance metrics were evaluated: MCC, F1score and Gmean. Results are presented in percentage. Non-evaluated results are marked as X.

Patient ID		MCC			F1score			Gmean		
		SE (%)	SP (%)	MCC	SE (%)	SP (%)	F1score	SE (%)	SP (%)	Gmean
**Population models**	P12	43	78	0.2	39	80	0.37	43	78	0.58
	P18	50	88	0.38	50	92	0.55	50	92	0.68
	P23	67	92	0.59	73	86	0.63	73	86	0.79
	P29	62	80	0.42	62	80	0.63	62	80	0.7
	P34	75	67	0.32	75	67	0.44	75	69	0.72
	P40	88	54	0.35	88	54	0.52	88	54	0.69
	P45	90	50	0.45	100	43	0.8	100	43	0.62
	P51	67	91	0.58	67	91	0.67	67	91	0.78
	P56	95	74	0.67	95	74	0.79	95	74	0.84
	P62	75	46	0.22	75	46	0.69	75	46	0.59
	**Median:**	**71**	**76**		**74**	**77**		**74**	**76**	
**Individualized models**	P12	71	50	0.23	67	50	0.37	65	60	0.61
	P18	88	75	0.54	73	100	0.4	78	50	0.55
	P23	73	60	0.22	75	100	0.5	69	67	0.68
	P29	67	62	0.24	56	67	0.57	70	75	0.75
	P34	83	50	0.29	91	50	0.5	91	67	0.78
	P40	X	X	X	X	X	X	X	X	X
	P45	80	100	0.73	67	75	0.67	50	75	0.5
	P51	X	X	X	X	X	X	X	X	X
	P56	83	67	0.31	86	67	0.67	62	67	0.65
	P62	75	75	0.45	75	69	0.76	75	67	0.63
	**Median:**	**77.5**	**64.5**		**74**	**68**		**69.5**	**67**	

**Table 3:** Median sensitivity (SN) and specificity (SP) of the model using support vector machines (SVM), excluding physical activity measures. Three different performance metrics were evaluated: MCC, F1score and Gmean. Results are presented in percentage. Non-evaluated results are marked as X.

Patient ID		MCC			F1score			Gmean		
		SE (%)	SP (%)	MCC	SE (%)	SP (%)	F1 score	SE (%)	SP (%)	Gmean
**Population models**	P12	33	75	0.08	42	75	0.37	42	75	0.56
	P18	64	82	0.4	45	92	0.5	45	92	0.65
	P23	73	84	0.51	73	85	0.61	73	85	0.79
	P29	56	75	0.31	56	70	0.58	56	70	0.63
	P34	77	65	0.32	77	65	0.45	77	65	0.71
	P40	86	63	0.39	86	63	0.52	86	63	0.73
	P45	86	69	0.56	86	69	0.84	86	69	0.77
	P51	67	82	0.44	67	82	0.44	67	82	0.44
	P56	90	69	0.56	85	69	0.71	85	69	0.76
	P62	63	65	0.28	63	65	0.66	63	65	0.71
	**Median:**	**70**	**72**		**69**	**73**		**69**	**73**	
**Individualized models**	P12	61	83	0.34	72	62	0.4	60	50	0.57
	P18	89	33	0.26	89	33	0.4	78	67	0.72
	P23	69	80	0.38	78	100	0.67	72	100	0.85
	P29	73	67	0.4	57	62	0.62	62	60	0.6
	P34	75	50	0.42	83	50	0.25	83	50	0.65
	P40	X	X	X	X	X	X	X	X	X
	P45	50	75	0.25	50	75	0.75	50	75	0.58
	P51	X	X	X	X	X	X	X	X	X
	P56	78	67	0.31	71	80	0.73	71	60	0.71
	P62	75	75	0.38	60	64	0.71	62	73	0.72
	**Median:**	**73**	**75**		**71**	**64**		**66.5**	**63.5**	

**Table 4:** Percentage of nights with hypoglycemia between 00:00 and 06:00. Results are expressed in percentages. The baseline simulation corresponds to the same simulation, but without rescue CHO.

		t = 20	t = 20	t = 40	t = 40	t = 60	t = 60
Patient ID	Baseline	Rescue CHO = 20	Rescue CHO = 30	Rescue CHO = 20	Rescue CHO = 30	Rescue CHO = 20	Rescue CHO = 30
P12	32.58	23.60	23.60	23.60	23.60	23.60	23.60
P18	30.34	19.10	19.10	20.22	19.10	21.35	21.35
P23	23.60	19.10	19.10	19.10	19.10	19.10	19.10
P29	32.58	23.60	23.60	28.09	26.97	29.21	28.09
P34	25.84	17.98	17.98	17.98	17.98	19.10	17.98
P40	35.96	22.47	22.47	22.47	22.47	23.60	22.47
P45	21.35	15.73	14.61	15.73	14.61	14.61	14.61
P51	38.20	24.72	23.60	25.84	24.72	24.72	24.72
P56	30.34	22.47	22.47	22.47	22.47	22.47	22.47
P62	34.83	21.35	21.35	25.84	21.35	28.09	26.97
**Median**	**31.46**	**21.91**	**21.91**	**22.47**	**21.91**	**23.03**	**22.47**

**Table 5:** Percentage of nights with nocturnal hypoglycemia (NH) between 00:00 and 06:00 for 20 and 30 g of rescue CHO (*p*-values < 0.0005).

	No Rescue CHO (Baseline)		Rescue CHO = 20 gr.		Rescue CHO = 30 gr.	
Patient ID	Nights with NH	# Hypos	#Hypos	Reduction	# Hypos	Reduction
	(%)			(%)		(%)
P12	32.58	29	21	27.59%	20	31.03%
P18	30.34	27	15	44.44%	16	40.74%
P23	23.6	21	13	38.10%	17	19.05%
P29	32.58	29	23	20.69%	21	27.59%
P34	25.84	23	20	13.04%	14	39.13%
P40	35.96	32	17	46.88%	17	46.88%
P45	21.35	19	15	21.05%	9	52.63%
P51	38.2	34	21	38.24%	22	35.29%
P56	30.34	27	20	25.93%	15	44.44%
P62	34.83	31	22	29.03%	21	32.26%
**Median**	**31.46**	**28**	**20**	**31.25%**	**17**	**36.76%**

**Table 6:** Statistical results of the percentage of time in different glycemic intervals (*p*-values < 0.05). Each blood glucose level interval is expressed in mg/dL. Abbreviations: TBR, time below range; TIR, time in range; TAR, time above range; NaN, not a number.

	Baseline	CHO = 20	Reduction	CHO = 30	Variation
**Below 54 (%)**	0.56 (0.48–1.33)	0.40 (0.17–3.15) *p* = 0.03	−35.09% (27–55%)	0.31 (0.23–3.60) *p* = 0.019	−44.44% (32–53%)
**Below 70 (TBR)**	7.24 (6.89–8.16)	4.89 (3.47–9.44) *p* < 0.001	−32.44% (27–56%)	4.34 (3.46–10.35) *p* < 0.001	−40.09% (30–57%)
**70–180 (TIR)**	92.51 (88.81–93.11)	94.56 (92.03–97.71) *p* = 0,39	+2.22%	94.19 (91.91–97.27) *p* = 0.44	+1.82%
**Above 180 (TAR)**	0 (0–4.59)	0 (0–13.96) *p* = 0.5	-	0.14 (0.01–30.46) *p* = 0.31	-

## Data Availability

Not applicable.
